# Endoscopic hemostasis with bipolar forceps coagulation for post-endoscopic sphincterotomy bleeding

**DOI:** 10.1055/a-2291-9399

**Published:** 2024-04-09

**Authors:** Haruo Miwa, Kazuya Sugimori, Kazuki Endo, Ritsuko Oishi, Hiromi Tsuchiya, Takashi Kaneko, Shin Maeda

**Affiliations:** 1 26437 Gastroenterological Center, Yokohama City University Medical Center, Yokohama, Japan; 2 Department of Gastroenterology, Yokohama City University Graduate School of Medicine, Yokohama, Japan

Post-endoscopic sphincterotomy (EST) bleeding is a well-known complication; however, an optimal procedure for endoscopic hemostasis has not been determined 1 . Electrocoagulation using bipolar forceps (Hemostat Y; Pentax, Tokyo, Japan) has been documented as effective for gastrointestinal bleeding while minimizing the risk of excessive tissue injury 2 3 4 ( Fig. 1 ). The bipolar forceps are available in both open and closed forms, and their soft and thin nature makes them compatible with the elevator system of a duodenoscope ( Fig. 2 ).

**Fig. 1:**
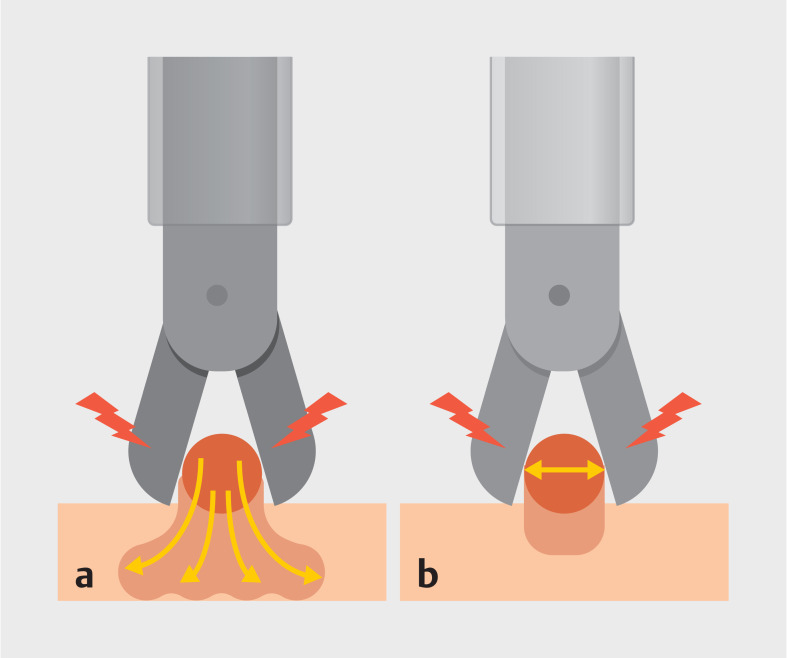
Endoscopic hemostasis with electrocoagulation. **a** Monopolar forceps hemostasis. **b** Bipolar forceps hemostasis.

**Fig. 2:**
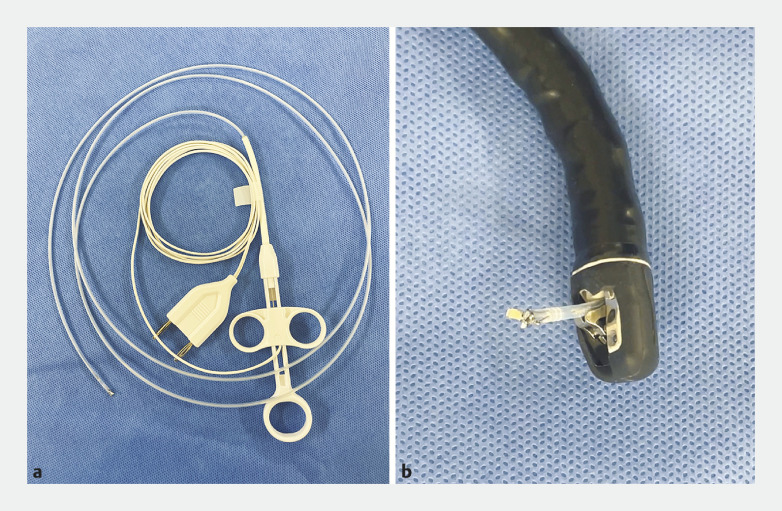
**a** Bipolar forceps (Hemostat Y; Pentax, Tokyo, Japan). **b** The soft and thin nature of the bipolar forceps makes them compatible with the elevator system of the duodenoscope.

A 78-year-old man with cholangitis underwent endoscopic transpapillary drainage, and EST was performed. Post-EST bleeding occurred on the third day; therefore, endoscopic hemostasis was performed by hypertonic saline epinephrine (HSE) injection and balloon compression ( Fig. 3 ). However, rebleeding occurred on the seventh day ( Fig. 4 , Video 1 ). During the emergency endoscopy using a duodenoscope (TJF-260; Olympus Medical Systems, Tokyo, Japan), spurting bleeding was observed from an exposed vessel behind the blood clot. A hemostatic clip was unsuitable as the bleeding point was located close to the orifice of the pancreatic duct. Attempts were made to grasp the vessel with bipolar forceps from the oral side; however, the oblique orientation of the papilla made hemostasis difficult. Despite coagulation with both the open and closed shape, pulsatile bleeding persisted. Subsequently, the bipolar forceps were inserted utilizing the elevator of the duodenoscope and the vessel was grasped. Finally, hemostasis was achieved, and HSE was injected to prevent rebleeding. No exposed vessels were observed in the post-EST ulcer 3 days later. Although bipolar forceps coagulation was repeatedly performed during endoscopic hemostasis, delayed perforation or pancreatitis was not observed.

**Fig. 3:**
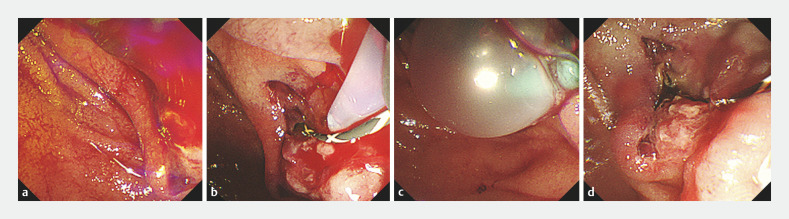
Endoscopic hemostasis on the third day. **a** Pulsatile bleeding was observed on the post-endoscopic sphincterotomy wound. **b** Hypertonic saline epinephrine was injected at the bleeding point. **c** Balloon compression was performed. **d** Endoscopic hemostasis was achieved.

**Fig. 4:**
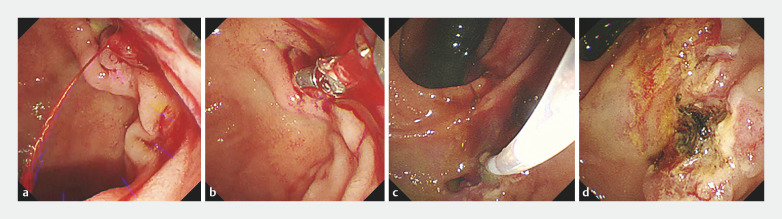
Endoscopic hemostasis on the seventh day. **a** Spurting bleeding was observed from an exposed vessel. **b** Electrocoagulation using the bipolar forceps was difficult due to the oblique orientation of the papilla. **c** Using the elevation system of the duodenoscope, effective coagulation was achieved. **d** Endoscopic hemostasis was successfully achieved by eliminating the exposed vessel.

Endoscopic hemostasis with bipolar forceps coagulation for post-endoscopic sphincterotomy bleeding.Video 1

To the best of our knowledge, this is the first report of a patient with post-EST bleeding undergoing endoscopic hemostasis with biopsy forceps coagulation. This technique was effective and feasible for spurting bleeding after EST.

Endoscopy_UCTN_Code_CPL_1AK_2AC

## Notes

Endoscopy E-Videos https://eref.thieme.de/e-videos
*E-Videos* is an open access online section of the journal *Endoscopy* , reporting on interesting cases and new techniques in gastroenterological endoscopy. All papers include a high-quality video and are published with a Creative Commons CC-BY license. Endoscopy E-Videos qualify for HINARI discounts and waivers and eligibility is automatically checked during the submission process. We grant 100% waivers to articles whose corresponding authors are based in Group A countries and 50% waivers to those who are based in Group B countries as classified by Research4Life (see: https://www.research4life.org/access/eligibility/ ). This section has its own submission website at https://mc.manuscriptcentral.com/e-videos .
